# The Genomic Signature of Demographic Decline in a Long‐Distance Migrant in a Range‐Extreme Population

**DOI:** 10.1111/mec.17805

**Published:** 2025-06-05

**Authors:** George Day, Thomas Bolderstone, Greg J. Conway, Tony Cross, Tony Davis, Matilda Dolan, Mervyn Greening, Colin Neale, Ian Nicholson, Kim Nicholson, Ann Ward, Nik Ward, Graeme Fox, Ewan Harney, Helen Hipperson, Kathryn Maher, Jamie Thompson, Rachel Tucker, Dean Waters, Kate L. Durrant, Terry Burke, Jon Slate, Kathryn E. Arnold

**Affiliations:** ^1^ Department of Environment and Geography University of York York UK; ^2^ NERC Environmental Omics Facility University of Sheffield Sheffield UK; ^3^ British Antarctic Survey Natural Environment Research Council Cambridge UK; ^4^ British Trust for Ornithology Norfolk UK; ^5^ University of Nottingham Nottingham UK

**Keywords:** European nightjar, genetic diversity, genomics, hDNA, museumomics, population genetics

## Abstract

Migratory birds are inherently vagile, a strategy that may reduce the impacts of habitat loss and fragmentation on genetic diversity. However, specialist resource requirements and range‐edge distribution can counteract these benefits. The European nightjar ( *Caprimulgus europaeus* ) is a long‐distance migratory bird and resource specialist. Like other long‐distance migrants, nightjar populations have declined across the British Isles and Northwestern Europe over the past century. With this decline well documented in the British Isles, there is a need to quantify its genetic impacts. We applied full genome resequencing to 60 historic (1841–1980) and 36 contemporary British nightjars. Nightjars exhibited a statistically significant 34.8% loss in heterozygosity and an increase in inbreeding over the last ~180 years, showing a departure from panmixia towards weak spatial structure in the modern population. Such fine‐scale structuring in migratory birds is rare. Our results provide a case study of fragmentation's impact on a species with specialist resource requirements at its range limit. Similar demographic declines in nightjars and other long‐distance migrants across Northern and Western Europe suggest that genetic patterns seen in the British population may reflect those in other nightjar populations and European avifauna. Whilst our results indicate no immediate conservation concern, they depict a trajectory of declining genetic diversity, increasing inbreeding and genetic structure, potentially shared with other migratory species. Our study highlights the value of applying spatiotemporal population genetics analysis to migratory birds, despite their inherent vagility.

## 1 Introduction

Numerous species and populations are under threat globally owing to ongoing habitat loss, degradation and fragmentation (Wake and Vredenburg 2008; Barnosky et al. 2011; Ceballos et al. 2015). Migratory birds are particularly vulnerable (Vickery et al. 2014; Bairlein 2016), with insectivorous species subject to severe population size reductions (Nebel et al. 2010, 2020; Sauer et al. 2017). Loss and fragmentation of habitat can drive population extinction risk by reducing connectivity and inhibiting dispersal (Frankham et al. 2010). Detrimental impacts are also recorded in vagile species with a perceived high tolerance to fragmentation, such as migratory birds (Lindsay et al. 2008; Hallworth et al. 2021; Larison et al. 2021). Reductions in population size and connectivity correspond with loss of genetic variation owing to reduced gene flow and the exacerbated effects of genetic drift (Frankham et al. 2010). Such genetic signatures may reflect a reduced capacity of a species or population to cope with environmental change and indicate a heightened extinction risk (Kempe 2008; Frankham et al. 2010). An understanding of the degree of differentiation among populations and levels of variation therein is important in delineating management units (Fuentes‐Pardo and Ruzzante 2017) and in determining population connectivity in difficult‐to‐monitor taxa, such as nocturnal and cryptic species (e.g., Crates et al. 2019; Larison et al. 2021).

An inability to sample populations before and after habitat loss and fragmentation leaves the impacts of these stressors on contemporary population genetic patterns difficult to quantify (Billerman and Walsh 2019). Museums provide a valuable resource (historic DNA; hDNA) for population geneticists to analyse time series data and perform temporal comparisons of contemporary and historic populations (Billerman and Walsh 2019; Fenderson et al. 2020; Irestedt et al. 2022). Typically, studies tracking spatiotemporal genetic structure have been restricted to model taxa, or geographically isolated, highly threatened species, for which the genomic indicators of demographic change are apparent (e.g., Feng et al. 2019; Robinson et al. 2021; Cavill et al. 2022; Westbury et al. 2022; but see Hansen et al. 2023; Kersten et al. 2023; Benham et al. 2024). In such cases, information on historic bottlenecks and contemporary population structure is imperative for effective conservation (e.g., translocation of individuals, delineating conservation units; Frankham et al. 2010). However, comparatively few temporal population genomics studies have been applied to non‐model taxa or species which have avoided severe bottlenecks or are distributed across a large geographical range (Payevsky 2006; Cox 2010). Consequently, the genomic footprint of this common demographic trend remains poorly understood (Lees et al. 2022; PanEuropean Common Bird Monitoring Scheme 2022), although recent studies have highlighted temporal genetic diversity decline in non‐model vagile species (Kersten et al. 2023; Benham et al. 2024).

Vagile species, such as long‐distance migratory birds, have the potential to negate the depletion of gene flow stemming from habitat loss and fragmentation because individuals are able to move between spatially distant breeding populations (Pârâu and Wink 2021). However, where mobile species rely on a spatial network of habitats or are habitat specialists, they may be susceptible to reductions in functional connectivity (Runge et al. 2014; Crates et al. 2019). Otherwise‐mobile species with high dispersal capabilities may then exhibit variation in population structure over small spatial scales (Morinha et al. 2017; Crates et al. 2019; Kimmitt et al. 2024). Populations at the extreme limits of a species' range may also be subject to reduced gene flow and are thus more likely to demonstrate increased structuring, inbreeding and lower genetic variation than central populations (Eckert et al. 2008). Habitat fragmentation and loss within range extremes may then have significant genetic consequences for threatened taxa, even in cases where species exhibit large geographic distributions or central population sizes (Fuller et al. 2007; Eckert et al. 2008; Runge et al. 2014). Despite their inherent vagility, migratory species remain vulnerable to genetic structuring among breeding populations. Apparent population admixture and panmixia shown in previous studies of migratory birds may be an artefact of using low‐resolution markers (Pârâu and Wink 2021). Indeed, historic conclusions of population admixture are likely to be reconsidered as Next Generation Sequencing (NGS) enables detection of fine‐scale structuring (Pârâu and Wink 2021), even in highly mobile migratory taxa (e.g., Larison et al. 2021; Kimmitt et al. 2024, but see Calderón et al. 2016; Pârâu et al. 2022).

**FIGURE 1:**
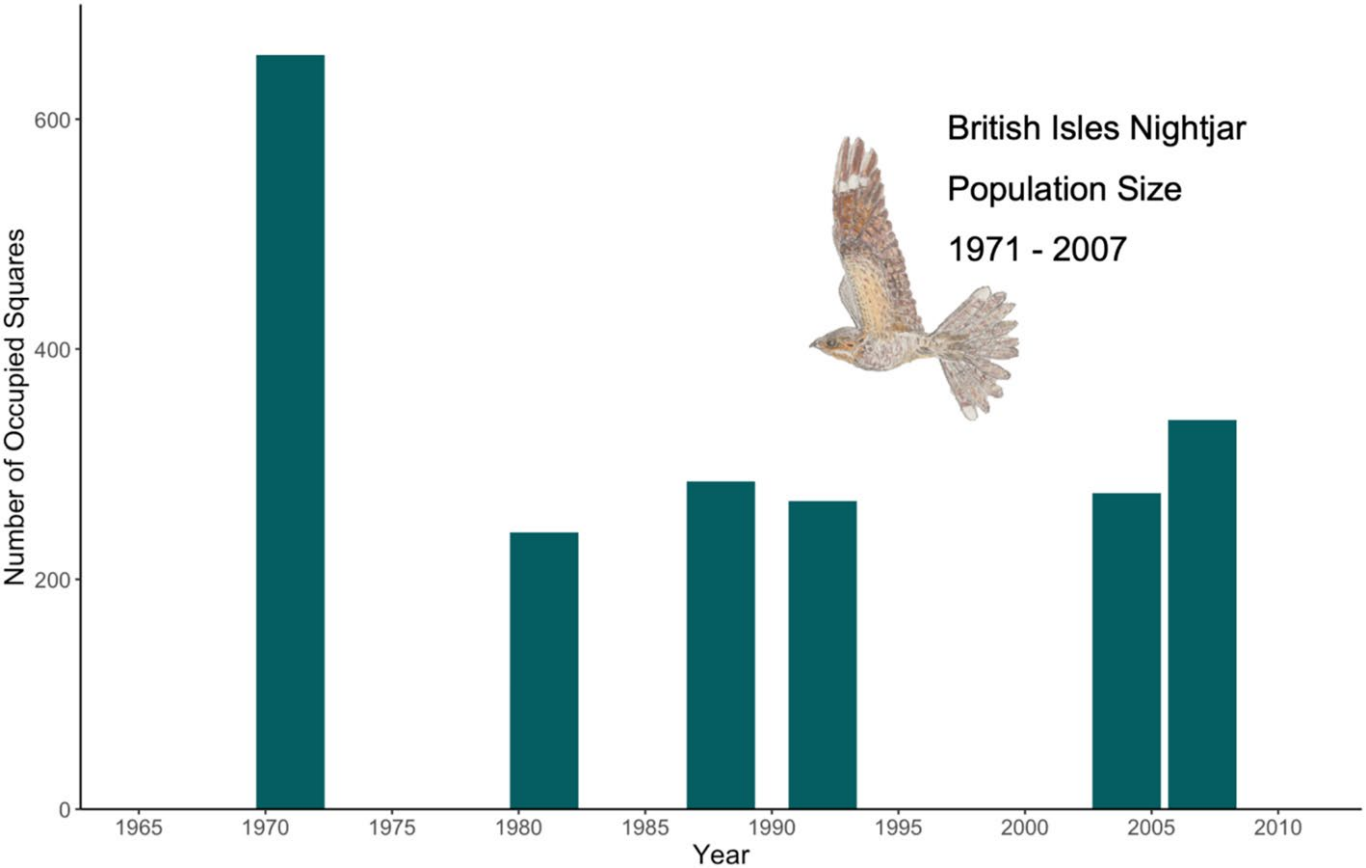
Actual change in the number of occupied 10 km squares (1971–2007) by nightjar across the entirety of Britain and Northern Ireland. Data derived from Sharrock (1976) Gribble (1983), Morris et al. (1994), Conway et al. (2007), Balmer et al. (2013).

**FIGURE 2:**
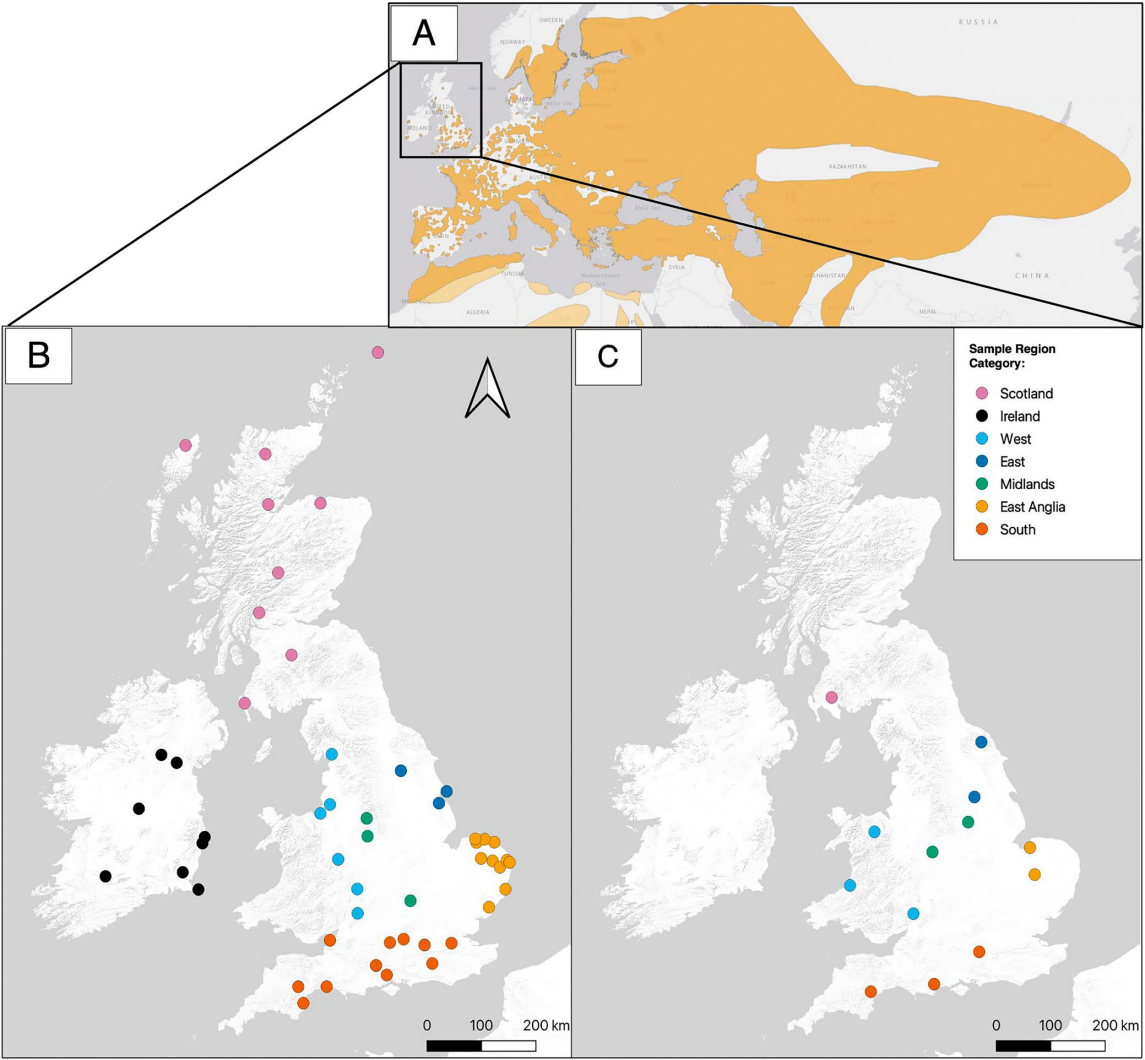
Modern breeding range map (A) and sampling locations of (B); historic and modern (C; *n* = 13 population centroids) nightjar samples. (A) Eurasian range map from (IUCN 2023), dark orange = breeding and light orange = found on passage migration only. (B, C) colours reflect assigned regions to each sample. Region classifications for each sample can be found in Table S1.

A long‐distance migrant, the European nightjar *Caprimulgus europaeus* (Cramp and Simmons 1985), henceforth nightjar, is a good study species to investigate the genetic signature of population decline in migratory birds, with the population decline and recovery well documented in the British Isles (Gribble 1983; Conway et al. 2007; Langston et al. 2007; Holloway 2010) at the species Western range limit (Cramp and Simmons 1985; Figure 2A). As recently as the 1800s, nightjars were a widespread breeding species across the entirety of the British Isles (Holloway 2010). The species underwent a population decline throughout the 20th century, undergoing *a* > 50% population reduction between 1966 and (Figures S1) 1981 (Figure 1). Range loss was most pronounced in the central and Western aspects of the species' British Range (Balmer et al. 2013), with nightjar declared extinct in Northern Ireland and near‐extinct in the Republic of Ireland in the late 20th century (Gribble 1983; Conway et al. 2007).

Nightjar are diet and habitat specialists, feeding predominantly on moths (Lepidoptera; Evens et al. 2020; Mitchell et al. 2022) and breed in heathland and felled plantation woodland (Conway et al. 2007). As such, degradation, loss and fragmentation of these habitats are one of the primary drivers of population declines (Langston et al. 2007). However, increased availability of felled coniferous plantations in the late 20th and early 21st centuries enabled a partial recovery in nightjar populations in Britain but not Ireland or the Republic of Ireland (Figure 1; Langston et al. 2007). Nevertheless, populations remain highly fragmented owing to the limited availability of suitable habitats (Langston et al. 2007). Ringing data suggest site fidelity (Cramp and Simmons 1985; Raymond et al. 2019) and philopatry in the species, which might reflect low connectivity and thus gene flow between breeding sites.

Species in the Caprimulgid family and other migratory nocturnal species are inherently difficult to study, owing to their cryptic and nocturnal nature (Crates et al. 2019; Larison et al. 2021). Quantifying the effects of habitat loss and fragmentation on population decline and connectivity in a hard‐to‐study and mobile species represents a significant challenge (Bi et al. 2013; Larison et al. 2021). In the British Isles, we have access to incomplete but relatively good quality data on nightjar including population demographic data to 1952 (Norris 1960) and access to museum samples going back to 1841. Thus, the British Isles population provides a good case study on the genetic signature of anthropogenic‐driven demographic decline in a migratory species breeding at an extreme range, with the pattern of population decline and fragmentation in the British population paralleling that of other threatened migratory species (PanEuropean Common Bird Monitoring Scheme 2022).

To assess the genetic signature of demographic decline in a range‐extreme population of a long‐distance migratory habitat specialist, we applied full genome resequencing to 96 individuals from both historic (*n* = 60 birds) and modern (*n* = 36 birds) populations, sampled over the historic and extant range of nightjar in the British Isles. Specifically, we aimed to characterise the spatiotemporal genetic structure in the historic (1840–1980) and modern (2019–2021) British population. We also aimed to determine whether this range‐extreme nightjar population demonstrated a change in global (genome‐wide) heterozygosity and runs of homozygosity (ROH) over time. We tested the hypothesis that there would be an overall decrease in heterozygosity and an increase in ROH, reflective of historic demographic decline in spite of recent partial recovery in the British nightjar population (Gribble 1983; Conway et al. 2007; Langston et al. 2007; Holloway 2010). Finally, we investigated whether global heterozygosity and ROH values varied among regions within temporal categories.

## 2 Materials and Methods

### 2.1 Modern Sample Collection and Study Sites

To provide DNA samples, buccal swab samples were collected between 2019 and 2021 from 33 nightjars across 13 breeding sites (Figure 2C) throughout the extant species' range with the help of citizen scientists (licensed British Trust for Ornithology bird ringers). Tissue samples were also obtained from three deceased birds, two from the ‘East’ region and 1 from ‘Scotland’. In total, samples from three individuals were selected per site (*n* = 36 across all sites), except for ‘North Wales’ (*n* = 1) and ‘Mid Wales’ (*n* = 2). For buccal cell sampling, nightjars were captured using mist nets within known breeding sites between June and September, to ensure only breeding or resident birds were sampled. Buccal swab samples were taken as per Day (2023). Tissue samples were taken from toe pads from dead nightjars (*n* = 3) and stored at −80°C.

### 2.2 Historic DNA Sample Collection

Nightjar skins collected between 1841 and 1980 were selected for sampling in order to span periods leading up to and encompassing the documented demographic decline throughout the 20th Century in the British Isles. Only skins with a known location of origin and dates were included, leaving a total of 60 individuals included in the study. An effort was made to sample from the complete historic British and Irish range (Figure 2B). Samples were taken from museum specimens by scraping the toe pad. A sterilised scalpel blade was used to remove a single 1–2 mm deep scrape of tissue from the toe pad of each nightjar skin (as per Sigurðsson and Cracraft 2014). Samples were then placed in a sterilised 1.7 mL Eppendorf tube and stored at room temperature prior to DNA extraction.

### 2.3 Sample Extraction and Library Preparation

#### 2.3.1 Sample Extraction

DNA from modern buccal swabs and tissue samples was extracted using a modified ammonium acetate method as per Day (2023) (see Appendix S1: Methods for a detailed account of extraction procedure). All historic samples were extracted using UV sterilised equipment and under a fume hood in a PCR product‐free laboratory to avoid contamination. For each toepad sample, the tissue was chopped into smaller pieces before being transferred to a 1.5 mL Eppendorf tube. Historic samples were extracted using a modified Qiagen Blood and Tissue kit protocol, with increased digestion stages (See Appendix S1: Methods for full extraction procedures).

#### 2.3.2 Library Preparation and Sequencing

All sample and library preparation post‐extraction was undertaken at the University of Liverpool NERC Environmental Omics Facility.

DNA libraries were prepared using the Mosquito platform with NEB Ultra II FS and NEB Ultra II DNA Kit protocols, depending on sample type (modern or historic). Libraries were indexed with unique dual indexes (IDT) and purified using AMPure XP beads. Library size and quality were assessed using the Qubit fluorometer and the Agilent Fragment Analyser. Sequencing was performed on the Illumina NovaSeq 6000 platform, generating 2 × 150 bp paired‐end reads. Full details of the library preparation protocol and sequencing can be found in Appendix S1: Methods.

### 2.4 Read Trimming and Alignment

Initial read trimming was undertaken using a custom pipeline by NERC Environmental Omics Facility Centre for Genomic Research. Briefly, Cutadapt (V 1.2.1; Martin 2011) was used to first trim all raw Fastq reads for the presence of Illumina adapter sequences. The option ‐O 3 was used, so that the 3′ end of any reads which matched the adapter sequence for 3 bp or more were trimmed. The reads were trimmed further using Sickle (V 1.33; Criscuolo and Brisse 2013) with a minimum window quality score of 20, reducing erroneous reads caused through the deamination of hDNA. Any reads shorter than 15 bp after trimming were removed. Read length and counts were characterised for both raw and trimmed reads (see Table S1).

Trimmed paired‐end reads were aligned against the European nightjar reference genome (Secomandi et al. 2021), using BWA Mem (V 0.7.1.7; Li and Durbin 2009). The resulting bam files were sorted using Samtools (V 1.17; Li et al. 2009) and PCR duplicates marked and removed using PICARD tools (V 3.0; Broad Institute 2023) ‘MarkDuplicates’. Finally, bam files were indexed using Samtools index (Li et al. 2009). Due to the variability in depth between modern (average depth: 8.4×) and historic samples (average depth: 5.3×), down sampling was performed on the trimmed modern reads to be used in downstream analysis where all samples were included. Down sampling was performed using Picard Tools ‘PositionBasedDownsampleSam’ (Broad Institute 2023). We randomly down sampled the modern reads by the proportional difference in the average number of reads between the modern and lowest depth historic samples (~71%) using the ‘FRACTION = 0.29’ command, down sampling the depth of the modern samples to 29% of their average depth (see Table S1). In total, reads from all 96 samples were successfully aligned to the nightjar reference genome (see Table S1).

### 2.5 Historic DNA Degradation

Historic samples can be characterised by postmortem substitutions (C to T and G to A) at the terminal ends of reads, owing to degradation associated with sample age and preservation methods (Briggs et al. 2007). These damage patterns can lead to the false identification of single nucleotide polymorphisms (SNPs) and thus have implications for downstream inferences. We used Mapdamage (V 2.2.1; Jónsson et al. 2013), with the default settings, to rescale the aligned reads (bam files) of the historic samples to account for base substitution at the terminal ends of reads. The program uses Bayesian estimation of the expected postmortem damage patterns to rescale the bam files, resulting in adjusted quality scores to account for the degradation. The resulting rescaled files were then used for all downstream analyses.

### 2.6 Genotype Likelihood Calling and Filtering

Owing to the low depth throughout, the samples used in this study uncertainty in genotype calls were accounted for by calling genotype likelihoods. A a software package developed for working with low‐quality, low‐coverage data, ANGSD (V0.938; Korneliussen et al. 2014), was used to produce the genotype likelihood scores for all individuals in the study. As per Çilingir et al. (2022), the GATK model (‘‐GL 2’) was used, and major and minor alleles inferred from genotype likelihoods (‘doMajorMinor 1’, ‘doMAF 1’). Only biallelic SNPs (‘‐skipTriallelic 1’) from properly paired and uniquely mapped reads (‘‐only_proper_pairs 1’ ‘‐uniqueOnly 1’) were retained. Further quality filtering was undertaken by discarding ‘bad’ reads (‘‐remove_bads 1’), as well as adjusting quality scores around indels (‘‐baq 1’) and for excessive mismatches (‘‐C 50’). Sites with a map and quality less than 30 and 20, respectively (‘‐ MinMapQ 30’ ‘‐minQ 20’), were also filtered out. Finally, sites with a polymorphism significance threshold of < 1e−6 were removed (‐SNP_pval 1e−6), and excess heterozygosity (> 0.5) were also filtered out to reduce potential paralogs.

Genotype likelihoods were successfully called (total n SNPS = 50,171,789, down sampled dataset = 42,413,393) for 94 individuals. Two samples, one modern and one historic, failed to produce genotype likelihoods. These were excluded from downstream analysis.

### 2.7 Population Genetic Analysis

#### 2.7.1 Data Filtering and Preparation

For all population genetic structure analysis, the genotype likelihoods were called as above with the addition of a minimum depth filter of one‐third the average depth (‘‐setMinDepth’), a maximum depth filter of ~3× average depth (‘‐setMaxDepth’) and a maximum missingness filter (‘‐minInd’) of 20% also applied. Owing to the large depth variation between samples, the depth characteristics of the historic samples were chosen to inform the filters used, with the minimum depth scaled as per the average depth of the historic samples. However, so as not to exclude a large proportion of the modern samples, the maximum was scaled as per the average modern sample depth (16×). The same filters were also applied to the down‐sampled dataset with the maximum depth reduced to 11x. Under the additional filters for the population genetics analysis, the full dataset contained a total of 1,144,436 SNPS with an average coverage of 4.2× for historic and 10x for modern samples. The down‐sampled dataset accounted for a total of 211,168 SNPS with an average coverage of 4.7×.

#### 2.7.2 Structure Analysis

To determine the patterns of spatiotemporal genetic structure, first patterns of genetic similarity among individuals were assessed using Principal Components Analysis (PCA) using PCAngsd (V 0.938; Korneliussen et al. 2014); this was run for all samples. Where clear structure was observed by PCA biplots, structure was investigated further by employing Bayesian clustering, Fixation Index (*F*_ST_), with patterns of isolation by distance (IBD) also tested.

**FIGURE 3:**
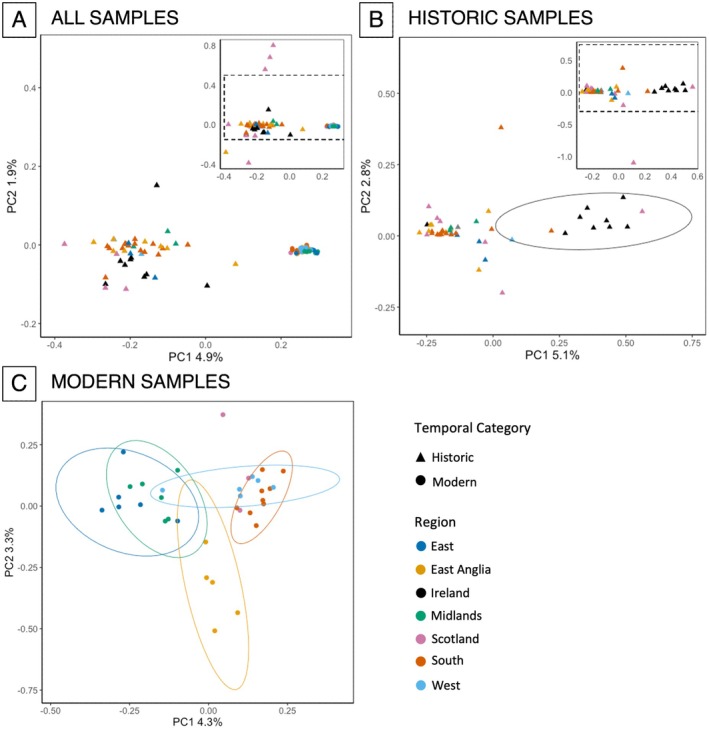
PCA biplots of genetic similarity. In all biplots, individuals with > 50% missingness (*n* = 15) have been removed from analysis. Plot (A) All (modern & historic samples), (B) Historic samples only and (C) Modern samples only. In the case of plots (A, B), the main plots are cropped subplots of the embedded plots (top right), which show all samples. The dashed boxes in the embedded plot show the cropped area presented in the main plot. The plot has been cropped to remove the effect of strongly differentiated individuals on interpreting the genetic structure. Where appropriate, regional groupings (coloured circles and triangles) are presented as 95% confidence ellipses.

PCA was run separately on (1) the full and down‐sampled datasets, as well as for (2) the historic and (3) the modern samples alone. PCAngsd produces a pairwise covariance matrix. This was exported to R (V 4.1.2; R Core Team 2020) to produce and visualise the principal components of the genotype data using the ‘eigen()’ command. PCA plots were then constructed using ggplot2 (V 3.4.1; Wickham 2016), plotted with 95% confidence ellipses to aid interpretation where appropriate. Two PCA were run for the historic samples, with and without g Irish samples, so to enable a direct comparison with the modern PCA results (See Appendix S1: Results Figure S5). Variation in missingness (missing SNPs), likely caused by differences in DNA quality between the down‐sampled modern and historic samples, appeared to drive clustering among temporal groups (see Appendix S1: Results Figure S2). To combat this, individuals from the historic sample pool with high missingness were removed from the PCA plots (> 50% missingness, *n* = 15 in full dataset, *n* = 13 in down sampled dataset), chosen arbitrarily as per (Whiting‐Fawcett 2024; Kumar et al. 2024). Notably, comparisons of applying a more stringent individual missingness filter (20%) showed that the chosen 50% threshold did not impact the clustering observed in the PCA biplot (see Appendix S1: Results Figure S3). Finally, the effect of outliers (possible migrants) on population structure were negated by presenting cropped PCA biplots for both aforementioned plots (Figure 3).

Where clustering of individuals was noted by PCA biplots, genetic structure was also determined using NGSAdmix (V 3.2; Skotte et al. 2013). To compare the levels of differentiation among regions, the *F*_ST_ was also calculated between region pairs, with evidence of IBD investigated. Full details of the NGSAdmix, *F*_ST_ and IBD analysis can be found in the Appendix S1: Methods.

### 2.8 Genome‐Wide Heterozygosity and Runs of Homozygosity

In order to investigate spatiotemporal changes in genomic diversity, genome‐wide autosomal heterozygosity, hereafter global heterozygosity, was calculated per individual (*n* = 94) in ANGSD using a folded SFS (‘‐dosaf 1’, ‘‐fold −1’), applying a minimum depth filter of 4× to reduce the effects of coverage on heterozygosity estimates (van der Valk et al. 2019). Heterozygosity analysis was conducted on all historic and down‐sampled modern samples, to reduce the effect of differences in sample depth. Average global heterozygosity (the number of singletons divided by the total number of sites) was calculated for each temporal category (historic and modern).

Temporal and spatial variations in global heterozygosity were assessed. To account for uneven sample sizes across regions, a mixed‐effect modelling approach was first used, including random slopes for year (time) by region classification. However, the mixed‐effect model did not successfully converge, and we encountered a singular fit. Variations in global heterozygosity among regions within each temporal category are non‐significant (One‐way ANOVA, *p* > 0.05 in both cases); temporal changes in heterozygosity were analysed using a linear regression model without the incorporation of region as a factor. Potential biases in temporal sampling were accounted for by weighting the global heterozygosity values based on temporal sampling intensity. We further assessed the robustness of the observed relationship between global heterozygosity and year by performing a randomisation test. This test involved permuting the global heterozygosity values and refitting the model 1000 times to assess the distribution of regression slopes. We compared the observed slope with this distribution to calculate a P‐value. Results were then plotted using ggplot2.

We estimated ROH using ROHan (Renaud et al. 2019). We only used samples with at least 5× coverage, which allowed us to test 16 historic samples and all 36 modern samples. ROHan was run only for autosomes and in ‘tvonly’ mode, which only considered heterozygosity at sites with transversions, not transitions (A↔G or C↔T), which are more prone to deamination in historic samples (Prüfer et al. 2010). ROH were estimated at two different heterozygosity thresholds: a ‘strict’ threshold where the proportion of heterozygous sites within a 1 Mb window was < 5 × 10^−5^, and ‘relaxed’, with this threshold set to 5 × 10^−4^. ROH were summarised as average segments in ROH (± standard error), together with the average length of ROH (± standard error), and genomic regions consistently in ROH across many samples were identified using bedtools multiinter (Quinlan and Hall 2010). Individual inbreeding coefficient (*F*_ROH_) within 100 kb windows was calculated per individual by dividing the proportion of ROH across the genome by 100 (Taylor et al. 2024). Variations in both *F*_ROH_ and lengths of ROH among regional categories were assessed using Kruskal Wallis and Dunns Post hoc tests and a one‐way ANOVA, respectively.

## 3 Results

### 3.1 Population Genetic Analysis

#### 3.1.1 Genetic Structuring Analysis of All Samples

Post‐missingness trimming, samples remained clustered in their temporal groups (Figure 3A), with little overall spatial structure evident. As such, the two temporal groups (Modern and Historic) were split and analysed separately (Figure 3B,C). However, with no other clear spatial or temporal clustering evident, further structure analysis was not applied to the full dataset.

#### 3.1.2 Genetic Structuring Analysis of Historic Samples

Where PCA was applied to the historic samples alone, the Irish samples formed a cluster compared with the remainder of the individuals from all other regions (Figure 3B). However, among the remaining individuals, there was little clear spatial or temporal structure. Upon removing the Irish samples from the analysis, similar patterns of panmixia among historic mainland British samples remained (Appendix S1: Results Figure S5). Notably, no clear temporal structure was observed among historic samples (Appendix S1: Results Figure S5); further structure analysis was then not applied.

#### 3.1.3 Genetic Structuring Analysis of Modern Samples

Where PCA was applied to the modern samples alone, weak spatial genetic structure was evident between regions, and samples could be broadly assigned to three main clusters (Figure 3C). Individuals from the West, South and from Scotland (far Northwest of the species' range) in the British Isles formed a tight group, except for a single Scottish outlier and a bird from Wales (Western region) which appeared to group with Eastern and Midland individuals (Figure 3C). The East Anglia birds accounted for the greatest differentiation across PC2, clustering together, although not as tightly as the West/Southern/Scottish individuals (Figure 3C). The remainder of the birds from the East and Midlands were grouped together, more tightly clustered than the East Anglia birds but less so than the South/West/Scottish birds. Bayesian clustering analysis highlighted that whilst the population might be weakly structured (best fitting *K* = 5, as per CLUMPAK; Appendix S1: Results Figure S6), admixture was present throughout all regions, suggesting moderately high gene flow among regions (Appendix S1: Results Figure S7), with *F*_ST_ values < 0.02 between all region pairs and only a weak IBD signature detected (Mantel test, *R* = 0.099, *p* > 0.3; see Appendix S1: Results Figure S7C).

### 3.2 Global Heterozygosity and Runs of Homozygosity

**FIGURE 4:**
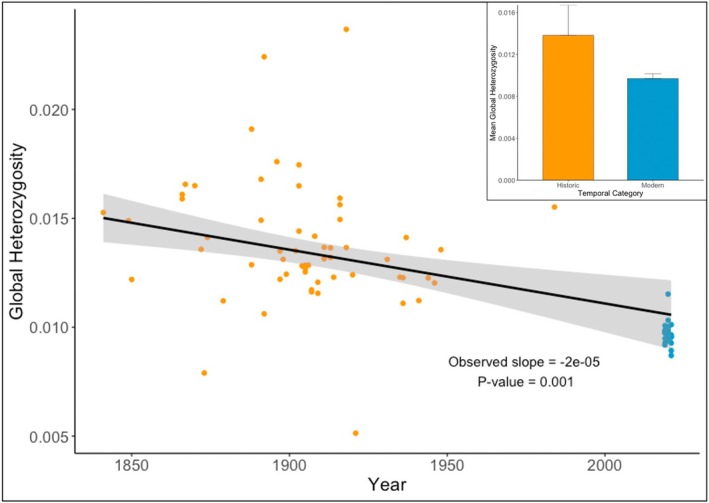
Weighted regression of global heterozygosity over time. The black line represents the weighted regression line, adjusted for sampling intensity across years, with 95% confidence intervals shown in grey. The observed slope and associated *p*‐value from the randomisation test are presented on the plot. Inset barplot shows differences in average global heterozygosity between the modern and historic samples, with error bars reflecting standard deviation. Throughout figure, orange = historic and blue = modern samples.

Global (genome‐wide) heterozygosity was determined for 94 individuals (59 historic and 35 modern samples). Weighted global heterozygosity was found to decline significantly over time, having reduced by 34.8% in modern samples, compared to historic samples (Figure 4). Notably, this decline was evident over the entirety of the timescale in which samples were collected, with heterozygosity appearing to decline throughout the 20th century (Figure 4). Global heterozygosity did not vary significantly among regional groups in either temporal category (One‐way ANOVA, *p* > 0.05 in both cases).

**FIGURE 5:**
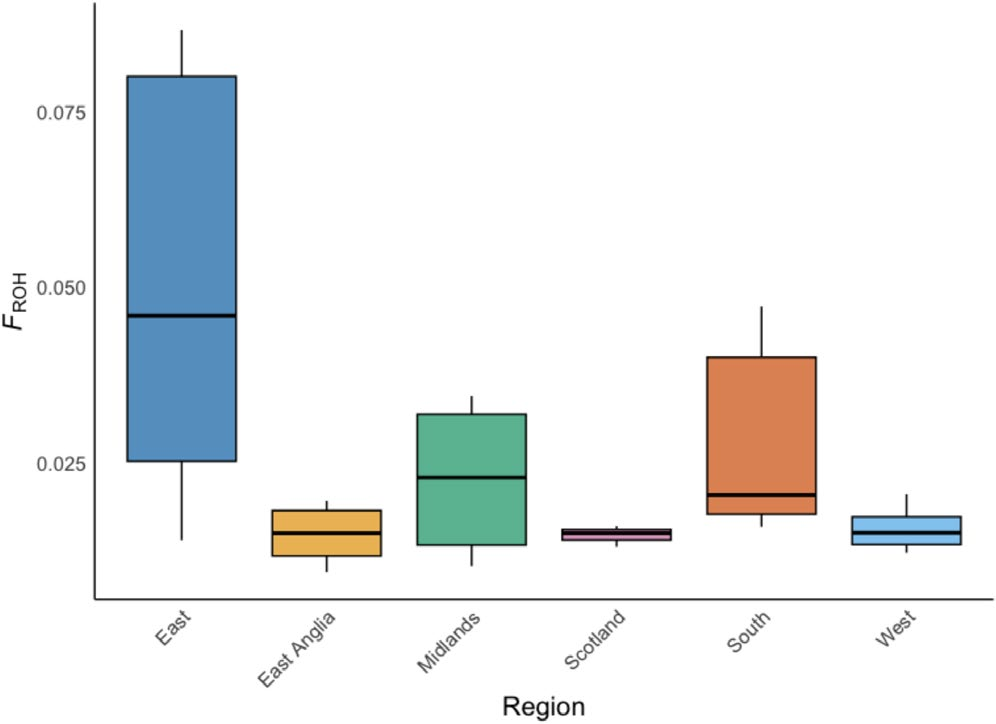
Average *F*_ROH_ within 100 kb windows across modern samples within each regional category. Boxes represent median (midline) first and third quartiles, and whiskers reflect value ranges.

We found no evidence for ROH in any of the 16 historic samples analysed at either the strict or relaxed thresholds. However, at the relaxed threshold, ROH were evident in all modern samples (Figure 5). Among the modern samples, an average of 27 Mb (± 3.4 Mb) were in ROH, corresponding to 2.51% (± 0.32%) of the autosomal genome. Modern samples featured between 3 and 11 ROH segments, which were on average 6.2 Mb in length (± 0.48 Mb), and we found two regions that were in ROH across all 36 samples: one on autosome OU015529.1 (40,000,001–44,000,000) and another on OU015531.1 (26,000,001–30,000,000). In both cases, ROH segments stretched to at least 4 Mb in all modern samples but reached up to 11 Mb and 17 Mb, respectively, in the most extreme cases.

In modern samples, *F*_ROH_ was found to vary significantly among regional categories (Kruskal–Wallis, χ^2^ = 11.86, df = 5, *p* = 0.0367; Figure 5), with the highest *F*_ROH_ found in the ‘East’ region (average *F*_ROH_ = 0.48), being significantly higher than all regions (Dunn's Test, *p* = < 0.05 in all cases), with the exception of ‘South’ (see Appendix S1: Results Table S2) (Figure 5). Notably, this elevated *F*_ROH_ in the ‘East’ region was likely driven by individuals from a single site (Humberhead Peatlands; average *F*_ROH_ = 0.62; Figure S7). Conversely, no significant variation in the length of ROH were detected among regional groups (One‐way ANOVA, *p* > 0.05).

## 4 Discussion

Between 1841 and 2021, the British Isles nightjar population exhibited a shift from complete panmixia among the historic samples (excluding Ireland) to weak regional structure in the modern population. Modern samples showed evidence of weak spatial genetic structure, broadly clustering into three regional groups. However, admixture was noted between all regions and only weak IBD was observed. Over the same timeframe, genomic diversity in this range‐extreme population underwent a significant and prolonged decline, with evidence of inbreeding increasing within the population and varying among regions in contemporary samples.

### 4.1 Weak Genetic Structure in the British Nightjar Population

Results from PSMC analysis suggest that nightjar likely show genetic structure across their European range (Day et al. 2024a, 2024b). However, on a fine scale, the vagility of birds often means that spatial structure is typically less likely than in more sedentary taxa (Coster et al. 2019; Pârâu and Wink 2021; Pârâu et al. 2022; Shephard et al. 2022). Indeed, the majority of migratory birds show little fine‐scale spatial genetic differentiation (reviewed by Coster et al. 2019; Pârâu and Wink 2021; Pârâu et al. 2022; but see Ralston et al. 2021; Shephard et al. 2022; Kimmitt et al. 2024). Despite this, nightjar showed evidence of weak fine‐scale structure in the modern population.

Nightjar are a habitat and resource specialist, breeding in heathland and plantation clear fell (Conway et al. 2007). Nightjar in the British Isles, as across much of their Western European range (Burfield and van Bommel 2004; Silvano and Boano 2012; BirdLIfe International 2022), exhibit a fragmented distribution, likely exacerbated by the loss of heathland throughout the 20th century (Conway et al. 2007; Langston et al. 2007). Aside from phylogenetic analysis (e.g., Mariaux and Braun 1996; Han et al. 2010; Larsen et al. 2007; Braun and Huddleston 2009; Schweizer et al. 2020) and ancient demographic reconstruction (Day et al. 2024a, 2024b), no population genetic data exist from nightjar or indeed other Caprimulgids, limiting phylogenetically relevant comparisons. Nevertheless, reductions in functional connectivity driven by fragmentation can drive genetic structure in otherwise vagile specialist species (e.g., Lindsay et al. 2008; Walsh et al. 2012; Pasinelli 2022), with many specialists exhibiting high breeding site fidelity and philopatry (Bech et al. 2009; Dolný et al. 2013; Camacho 2014; Byer and Reid 2022; but see Coster et al. 2019), including nightjar (Vilella 1995; Wilkinson 2009; Camacho 2014; McGuire et al. 2021). High philopatry and reductions in functional connectivity over the past century may go towards explaining the change from panmixia to weak structure in the mainland British nightjar population. Moreover, contemporary genetic structure in the British population may also be exacerbated by the position of the British Isles at the species' range extreme, owing to uni‐directional or reduced geneflow from the species' range centre (Schwartz et al. 2003; Langin et al. 2017).

Whilst no significant variation in *F*_ST_ was found among regions, our analysis suggests weak clustering of the South, Scottish and Western populations, East and Midlands populations and East Anglia into three groups. With no clear IBD or significant geographic barriers between these regional groups, reasons for this weak clustering are not immediately evident. Moreover, migratory connectivity may go towards explaining the spatial pattern of weak structure. Following recent insights into nightjar migration, birds breeding in East Anglia tended to return to breeding sites via Southeast England, reducing the chance for mixing with Western or the Southern populations sampled here (Lathouwers et al. 2022). Birds breeding in Wales (West) returned to breeding sites via the South of England, taking the shortest sea crossing (Lathouwers et al. 2022), providing the opportunity for the mixing observed in this study. Although no migration tracking data currently exist for Scottish, East or Midland populations, it is expected that those birds migrating to distant locations, away from southeast England, will have greater opportunities to mix with local breeding birds en route.

### 4.2 Spatiotemporal Changes in Heterozygosity and Runs of Homozygosity

The shift from panmixia towards weak genetic structure in the British nightjar population was accompanied by a significant 34.8% loss in genome‐wide heterozygosity between 1841 and present.

In addition to the loss of heterozygosity, we found a stark contrast in ROH between the two temporal categories, with ROH seemingly absent in the historic population but present within all modern samples. These changes coincided with a large reduction in population size over the last ~120 years (≥ 50%), although the population has since shown partial recovery throughout the late 20th century (Conway et al. 2007; Langston et al. 2007). Our results highlight that, despite this, the population bears a signature (loss of heterozygosity and recent inbreeding) of the historic changes in population size, likely driven by habitat loss and fragmentation (Langston et al. 2007). However, the decline in heterozygosity shown here begins > 100 years prior to the documented demographic decline of nightjar in Britain. Indeed, the true extent of population decline in nightjar over the last 200 years, as in most species, is unknown owing to the paucity of accurate census data. Taking global heterozygosity as a population size proxy (Grundler et al. 2019), our data suggest that the decline of nightjar in Britain was likely underway prior to the documented significant losses during the 20th century. With industrialisation throughout the 19th century (Allen 2004), and significant forest clearance prior to that (Simmons et al. 2021), anthropogenic land use change has likely been driving historic population reduction in the species for a number of centuries. This trend can likely be expanded to other Western European nightjar populations which have shown similar demographic change (Burfield and van Bommel 2004; BirdLIfe International 2022) and patterns of heathland loss and national industrialisation over the last 200 years (Webb 1998; Piessens et al. 2005). Habitat loss and degradation across the species' migratory routes and wintering grounds may also have contributed to the changes observed in our study. Like other long‐distance migrants, nightjars are exposed to stressors throughout their annual geographic range (Newton 2010; Hewson et al. 2016; Howard et al. 2020). Additionally, climate change‐driven factors, such as phenological asynchrony, increasingly exacerbate these challenges for long‐distance migratory insectivorous birds, including nightjars (Gilroy et al. 2016).

The impact of habitat loss and fragmentation on population size, and ultimately heterozygosity, was likely intensified by the British population being at the periphery of the species' range (Pironon et al. 2017; Perrin et al. 2021; Frantz et al. 2022). Following the central‐margin hypothesis, populations at the range limits of a species tend to exhibit lower genetic diversity than central populations, as gene flow decreases towards the periphery, thereby amplifying the effects of genetic drift (Lesica and Allendorf 1995; Eckert et al. 2008). This pattern can also occur at smaller scales, as demonstrated by Langin et al. (2017), who found reduced heterozygosity in marginal populations of the Island scrub‐jay ( *Aphelocoma insularis* ) over distances greater than 20 km. In our study, although significant variation in heterozygosity was not observed among regions in either the historic or modern samples, *F*_ROH_ did vary significantly across regions in the modern samples, likely reflecting region‐specific levels of inbreeding. This variation is consistent with reduced gene flow among modern samples, as indicated by our structuring analysis. Notably, *F*_ROH_ also differed between specific breeding sites, with the high *F*_ROH_ values in the ‘East’ region appearing to be driven by the three individuals sampled from the Humberhead Peatlands in East Yorkshire. However, the small sample size (*n* = 3 individuals per site) limits further investigation of site‐specific trends. The reasons for region‐ and site‐specific differences in inbreeding are not immediately clear, as region‐specific heterozygosity values did not vary significantly and pairwise *F*_ST_ were low in our study. Nevertheless, differences in inbreeding among regions and breeding sites are likely linked to variation in functional connectivity, habitat quality and local population trends (Frankham et al. 2010), possibly exacerbated by the range‐extreme position of the British nightjar population (Eckert et al. 2008).

### 4.3 Implications for Conservation and Conclusions

The 34.8% loss of global heterozygosity and increase in inbreeding (*F*_ROH_) reflects the genomic impact of demographic decline and spatial fragmentation in the British nightjar population. However, whilst the loss of genomic diversity is significant, nightjar global heterozygosity in the modern British population remains high compared with threatened avifauna internationally (e.g., average global heterozygosity rate in nightjar = 0.00969, in Seychelles magpie‐robin *Copsychus sechellarum* = 0.00015, see also Cavill et al. 2022; Wang et al. 2022), presenting no immediate causes for concern. Furthermore, whilst we have highlighted a temporal increase in inbreeding in nightjar, the lack of ROH observed at our strict threshold likely reflects only a low level of inbreeding within the modern population and also presents no immediate cause for concern. Nevertheless, our results show that despite the recent partial recovery, the effects of demographic decline in the British population are not negligible. Rather, the trend in heterozygosity and inbreeding likely reflects a long‐term, ongoing decline in population size and genomic diversity. This temporal trend in heterozygosity and inbreeding may have been driven by habitat loss and fragmentation, with our genetic structuring results seemingly corroborating this, showing a shift from panmixia to weak regional level structuring over the last ~180 years. Similar trends have also been found in other studies where a temporal sampling strategy has been employed, with these trends also linked to anthropogenic habitat loss and fragmentation (Feng et al. 2019; Vandergast et al. 2019; Robinson et al. 2021; Ericson et al. 2022; Westbury et al. 2022; Kersten et al. 2023; Benham et al. 2024). Whilst the spatial structure in the modern nightjar population is weak, the temporal change from complete admixture towards regional differentiation is notable, with regional variation in inbreeding also evident. The underlying causes of gene flow resistance (i.e., dispersal constraints including landscape features, habitat connectivity, natal philopatry, individual quality; Holderegger and Wagner 2008; Camacho et al. 2013) among regions are not immediately obvious at this time, and characterising these may prove informative for future conservation measures for nightjar in Britain.

Our study demonstrates the genomic signature of population decline in a long‐distance migratory bird at its range extreme. We add to a growing body of evidence, showing that species with a high dispersal potential may also bear the genomic signature of population decline (Kersten et al. 2023; Benham et al. 2024; Kimmitt et al. 2024), emphasising the role of resource specialisation in mediating a species response. The demographic decline exhibited by nightjar is not unique, with significant reductions in population size also recorded across a number of migratory birds and resource specialists (Bairlein 2016). The combination of high‐resolution analysis and temporal sampling enables accurate insight into the extent and impacts of population decline on contemporary genetic and demographic patterns. This approach provides a valuable opportunity to quantify the effects of anthropogenic habitat destruction and fragmentation in present‐day populations.

## Author Contributions

G.D., K.E.A., T.B. designed the study, with input from D.W. and K.L.D. Fieldwork was conducted by G.D., G.J.C., A.W., T.C., M.D., T.D., N.W., T.B., I.N., C.N., M.G. and K.N. When not sampled by museum curators, toe and footpad scrapes were sampled by G.D. Lab work was conducted by G.D., R.T. and J.T. Downstream bioinformatics was undertaken by G.D., with assistance from G.F., H.H. and K.M. Runs of homozygosity analysis was performed by E.H. Data analysis was undertaken by G.D., with assistance from K.E.A., D.W. and J.S. Manuscript preparation and writing was completed by G.D., with K.E.A., K.L.D., T.B., J.S., D.W., E.H. and G.C. assisting with the initial review. Finally, all the authors read and contributed to the manuscript draft prior to submission.

## Ethics Statement

All work conducted in this study was reviewed and approved by the University of York's Animal and Welfare Ethical Review Body. DNA collection via buccal swabs was reviewed and approved by the British Trust for Ornithology (BTO) special methods technical panel, with accredited agents trained appropriately and possessing C or A class bird ringing permits from the BTO. Bird handling and ringing were conducted following best practices outlined in Redfern and Clark (2001).

## Conflicts of Interest

The authors declare no conflicts of interest.

## Supporting information


Appendix S1.

**Tables** **S1‐S3.**

## Data Availability

All raw sequence data used in this study are freely available from the GenBank database under BioProject: PRJNA1162521 (Day et al. 2024a).
