# Supplementary material for: The Genomic Signature of Demographic Decline in a Long‐Distance Migrant in a Range‐Extreme Population
Source: Mol Ecol. 2025 Jun 5;34(13):e17805. doi: 10.1111/mec.17805 (PMC12186721; doi:10.1111/mec.17805)
Supplement: Supplementary file 1 — Appendix S1. [file MEC-34-e17805-s002.docx]

**Supporting Information**

# **The genomic signature of demographic decline in a long-distance migrant in a range extreme population**

George Day^1,2,3*^, Thomas Bolderstone1^1^, Greg J Conway^4^, Tony Cross1^1^, Tony Davis1^1^, Matilda Dolan1^1^, Mervyn Greening1^1^, Colin Neale1^1^, Ian Nicholson1^1^, Kim Nicholson1^1^, Ann Ward1^1^, Nik Ward1^1^, Graeme Fox^3^, Ewan Harney3, Helen Hipperson^3^, Kathryn Maher^3^, Jamie Thompson^3^, Rachel Tucker^3^, Dean Waters1^1^, Kate L Durrant^5^, Terry Burke^3^, Jon Slate^3^, Kathryn E. Arnold1^1^

**Supplementary Methods**

**Sample DNA extraction and quantification**

*Modern sample*s – Buccal swabs were removed from storage tubes and placed into 1.5ml microcentrifuge tubes before being cut down using sterile scissors, leaving ~5cm of swab stalk remaining above the tip. Tissue samples were chopped into smaller pieces (2mm^2^ cubes) before being transferred to a 1.5ml microcentrifuge tube. Per tube 250µl of Digsol buffer and 10mg/ml 10µl of Proteinase-K (20mg/ml for tissue samples) were added before vortexing of 2.5 minutes and a 24 hr digestion stage at 55°C in a rotating oven (12hr for tissue samples). After incubation a 300µl of Ammonium acetate was added before vortexing for 20 minutes in order to precipitate the proteins. Buccal swabs were then removed from the microcentrifuge tubes and discarded. The remaining solution in the Eppendorfs was then centrifuged for 10 minutes at 13,000, and the supernatant aspirated into a clean 15ml microcentrifuge tube. 1ml of 100% ethanol was added to the solution and Eppendorfs inverted 12 times to precipitate the DNA. Samples were further centrifuged for 10 minutes at 13,000 rpm and ethanol poured off. To rinse the pellet 500µl of 70% ethanol was added to the sample and Eppendorfs again inverted 12 times. A final centrifuge step (5 minutes at 13,000rpm) was used to ensure no DNA loss when pouring off the ethanol. Samples were finally left to air dry for 1 hour and 40µl of LowTE added. Samples were then placed in a water bath for 30 minutes (37 °C) to ensure pellet resuspension, before being stored at -20°C prior to quantification.

*Historic Sample DNA Extraction* – A small amount of Buffer ATL was added to a sterile microscope slide before carefully cutting up the toepad sample in small (≤ 1mm^2^) chunks. The sample was then pipetted into 1.5ml Eppendorf, before adding 180µl of Buffer ATL and 20µl of 1M DTT and 20µl 10mg/ml Proteinase-K. The solution was then vortexed thoroughly before being placed in a rotatory incubator at 56 °C overnight. The sample was then pipetted into 1.5ml Eppendorf, before adding 180µl of Buffer ATL and 20µl of 1M DTT and 20µl 10mg/ml Proteinase-K. The solution was then vortexed thoroughly before being placed in a rotatory incubator at 56 °C overnight. Upon removing the sample from the incubator samples were vortexed again for 15 seconds. At this stage if samples had not appeared to be digested, more Proteinase-K (10mg/ml) was added and the incubation stage performed again. Buffer AL (200µl) was then added before vertexing again and further incubated at 70°C for 10 minutes. Then 200ul of 100% ethanol was added to the solution before vertexing thoroughly. A spin-column was then inserted into a 2ml collection tube before pipetting the entire mixture through the spin column and centrifuges at 9000 rpm for 1 minute. The spin column was then placed in a new collection tube and 500ul of Buffer AW2 added through the column before centrifuging again at 13000 rpm for 4 minutes. The spin column is then places in a 1.5ml eppendorf before eluting the DNA by washing with 40ul Buffer AW, being careful to ensure the buffer makes direct contact with the spin column membrane. The sample is centrifuged again at 8000rpm for 1 minute. Finally, the previous step was repeated by running the 40µl resultant solution back through the column. This helped to maximise the final DNA yield. The final DNA, which was suspended in 40µlof buffer AW was then frozen at -20°C prior to quantification, library preparation and sequencing.

**DNA Quantification**

All modern samples (n = 30) were quantified using a using a FLUROstar Optima Spectrophotometer (BMG Labtech). BMG black plate wells were loaded with 2µl of each sample and 7 calf thymus quantification standards of 0, 3.24, 6.49, 12.98, 25.95, 51.9 and 103.8ng/µl. Hoesct dye (200ul) was added to each well before being run on the fluorometer and readings taken. All samples which exceeded 10ng/ul were diluted to 10ng/µl concentration with Low T.E prior to genotyping. Samples with a DNA concentration below 10ng/µl samples were not dehydrated to increase DNA concentration owing to the low elute (30 µl). Historic samples (n = 60) were quantified individually using a Qubit fluorometer.

#### **Quality analysis**

To test whether the age and collection of samples (hDNA) affected sample yields a one-way ANOVA was used to test the hypothesis that both sample age and collection of origin would significantly affect the final DNA yield from hDNA samples. Modern samples were extracted with an average yield of 14.37 ng/µl (stdev = 23.39 ng/µl), whilst historic samples were extracted with a lower average yield of 6.58 ng/ µl (stdev = 10.27 ng/µl), and four samples failing to produce a reading suggesting very low (<0.1ng/µl) yields (Table S1. Whilst yield declined with sample age, sample age alone did not significantly predict yield (one -way ANOVA, F_(1,46)_ = 0.404, P >0.5) (Fig S1). However, the identity of the museum collection from which samples originated was found to significantly predict yield (one -way ANOVA, F_(7,46)_ = 15.52, P < 0.001), with three collections (BM, NMS and YMT) accounting for notably elevated yields compared to the remainder of collections, even whilst controlling for sampling year (collection year * collection ID, F_(6,46)_ = 0.135, P > 0.1).

**Library Preparation for Sequencing**

Owing to the differences in yield and inherent degradation and fragmentation associated with hDNA (Billerman and Walsh, 2019; Irestedt et al., 2022) the historic and modern samples were prepared for sequencing separately.

*Modern samples –* Modern samples were normalised for a total library input of 20ng. The standard in-house protocol for 1/10th miniaturised NEB Ultra II FS for Mosquito platform was followed, with a fragmentation time of 6 mins and 12 cycles of PCR. Libraries were indexed using unique dual indexes (IDT) and purified with a final 0.6x AMPure XP bead clean. Completed libraries were quantified using Qubit (Thermo Fisher) and fragment sizes assessed using the Fragment Analyzer (Agilent).

*Historic samples –* Historic samples were prepared without fragmentation using the NEB Ultra II DNA Kit. Up to 100ng per sample was used as input, where available. The protocol was followed according to the kit manual, without the optional size selection step due to the small insert size. The samples were indexed using 12 cycles of PCR to match the library prep for samples prepared using the mosquito, using unique dual indexes (IDT) and purified with a 0.9x AMPure XP bead clean. The concentration of the final libraries was assessed using Qubit and the sized using the Fragment Analyzer (Agilent) and equimolar pooled. The final pool was size selected using the Pippin Prep, selecting for 180-600bp on a 2% gel. The size selected pool was purified with a 1:1 AMPure XP bead purification and eluted in 20ul nuclease free water. The final concentration and library size was assessed using the Agilent Bioanalyzer.

The quantity and quality of the pool was assessed by the Bioanalyzer and subsequently by qPCR using the Illumina Library Quantification Kit from Kapa on a Roche Light Cycler LC480II according to manufacturer's instructions. Briefly, a 10µl PCR reaction (performed in triplicate for each pooled library) was prepared on ice with 8µl SYBR Green I Master Mix and 2µl diluted pooled DNA (1:1000 to 1:100,000 depending on the initial concentration determined by the Qubit® dsDNA HS Assay Kit). PCR thermal cycling conditions consisted of initial denaturation at 95°C for 5 minutes, 35 cycles of 95°C for 30 seconds (denaturation) and 60°C for 45 seconds (annealing and extension), melt curve analysis to 95°C (continuous) and cooling at 37°C (LightCycler® LC48011, Roche Diagnostics Ltd, Burgess Hill, UK).

Following calculation of the molarity using qPCR data, template DNA was diluted to 300pM and denatured for 8 minutes at room temperature using freshly diluted 0.2 N sodium hydroxide (NaOH) and the reaction was subsequently terminated by the addition of 400mM TrisCl pH=8. To improve sequencing quality control 1% PhiX was spiked-in.

The libraries were sequenced on the Illumina® NovaSeq 6000 platform (Illumina®, San Diego, USA) following the standard workflow over 1 lane of an S5 flow cell, generating 2 x 150 bp paired-end reads.

#### **Admixture Analysis**

####

NGSAdmix deals with genotype likelihood input files and operates similarly to the Bayesian clustering software STRUCTURE. NGSadmix assigns individuals to clusters based on genetic similarity, aiming to minimise variation among individuals within each cluster. NGSAdmix assumes independence of loci, thus linked loci should be filtered prior to analysis. Here we used NGSld (V 3.2; Fox et al., 2019) to first perform linkage analysis and secondly prune the SNP’s used by NGSADMIX. Linkage analysis was performed on the Beagle file generated from the full dataset after the filters used for the population genetics analysis were applied (see Main Text). Pairwise LD were calculated and linked loci were pruned, allowing for a maximum among-SNP distance of 100kb and a minimum weight (LD estimate between two SNPs) of 0.5. NGSAdmix was then applied to the pruned data. NGSAdmix was run for the modern samples only, owing to little spatial structure evident among the historic samples. The software was run for each SNP set with cluster (*K*) 2 - 10, performing 10 replicates per run. The results of the analysis were then visualised in R, with the optimum *K* value determined for each SNP set using CLUMPAK (Kopelman et al., 2015).

#### **Fixation Index (F_ST_)**

To compare levels of differentiation between regions the fixation index (*F*_ST_) was calculated between each region pair. As with admixture analysis, regional differentiation was assessed in the modern samples only. Weighted pairwise *F*_ST_ values were calculated between regions (n = 6). *F*_ST_ values were calculated in ANGSD and realSFS. Firstly, site allele frequency (SAF) likelihood values were estimated for each site/region from the genotype likelihoods (‘-doSAF 1’) calculated as per the geno0mewide heterozygosity analysis (see Main Text), with the reference genome used in place of the ancestral sequence. The spectra were then used to calculate a pairwise folded site frequency spectra (SFS) between each population/region pair in realSFS. The global pairwise weighted *F*_ST_ values were then calculated in realSFS using ‘-fst stats’ and exported to R for visualisation in a heat map using ggplot2.

#### **Isolation by distance**

A pattern of isolation by distance (IBD) among modern samples was tested by correlating genetic distance (*F*_ST_) between regions, as calculated above, with Euclidean distance between breeding site and region centroids. Euclidean distances were calculated in QGIS (V 3.30.0 QGIS Association, 2023). A Mantel test was used to test the correlation between the genetic and euclidean distance matrices, testing the null hypothesis that population/region *F_ST_* would not increase with Euclidean distance. Analysis was conducted in R.

**Supplementary Results**

**DNA Yields**

Modern samples were extracted with an average yield of 14.37 ng/µl (stdev = 23.39 ng/µl), whilst historic samples were extracted with a lower average yield of 6.58 ng/ µl (stdev = 10.27 ng/µl), and four samples failing to produce a reading suggesting very low (<0.1ng/µl) yields (Table S1). Whilst yield declined with sample age, sample age alone did not significantly predict yield (one -way ANOVA, F_(1,46)_ = 0.404, P >0.5) (Fig S1). However, the identity of the museum collection from which samples originated was found to significantly predict yield (one -way ANOVA, F_(7,46)_ = 15.52, P < 0.001), with three collections (BM, NMS and YMT) accounting for notably elevated yields compared to the remainder of collections (Fig S1), even whilst controlling for sampling year (collection year * collection ID, F_(6,46)_ = 0.135, P > 0.1).

**Admixture Analysis**

Admixture analysis of the modern samples showed that whilst the population might be weakly structured (best fitting *K* = 5, as per CLUMPAK; Fig S6), admixture was present throughout all regions, suggesting moderately high gene flow among regions (Fig S7A). Nevertheless, at *K* = 4, the proportion of shared ancestry appeared similar within the East/Midland and West/South regions respectively, with clustering sharing some congruence with that suggested by the PCA biplot (Fig 3C; Fig S7A). Notably, individuals within East Anglia showed less admixture than other regional categories (Fig S7A), this may be congruent with the clear segregation of East Anglia birds evident from the PCA (Fig 5), this was particularly clear at *K* = 5 (Fig S7A). However, it is clear that significant admixture remained among all regions (Fig S7A), as typified by the high variation in Delta K and minimal difference between the two highest delta *K* values (CLUMPAK DeltaK; *K* 3 = 1.058, *K* 5 = 1.105; Fig S6).

**Fixation Index Analysis**

*F*_ST_ values were calculated between regions for the modern samples alone. Individuals showed low levels of differentiation across regions, with weighted pairwise *F*_ST_ ranging from 0.002 - 0.018 (Fig S7B). *F*_ST_ values were largely congruent with the PCA results (Fig 3C), with the exception of the Scottish birds (Fig S7B). The lowest *F_ST_* values occurred between the West and South and East and Midland regions respectively (Fig S7B), suggesting similar, although weak, clustering to that in Fig 3C. With the exception of Scotland, the higher levels of differentiation were found mostly between East and South/West regions as per the clustering suggested in Fig 3C. East Anglia showed moderate differentiation from all regions, again with the exception of Scotland. The only regional *F*_ST_ values not congruent with the PCA concerned the Scottish individuals (Fig 3C; Fig S7). The anomalous results may be due to the small sample size (n = 3 individuals from 1 site), whilst the average number of individuals contributing to the other regions was six. Small sample sizes are known to skew *F*_ST_ results (i.e. < 3; Willing et al., 2012), although extra steps were taken in my analysis to remove the effects of sample size (i.e. inclusion of ‘-which fst’ command) as much as possible. The single highly differentiated Scottish individual shown in Fig 3C may further account for the high pairwise *F*_ST_ values concerning the Scottish region. However, as with the PCA, increments of differentiation presented here are low, with inter-region variation in pairwise *F*_ST_ values occurring within a small range. Finally, IBD analysis showed a weak but non-significant positive relationship between genetic (*F*_ST_) and geographic (km) distances (Mantel test, R = 0.099, P > 0.3; Fig S7C).


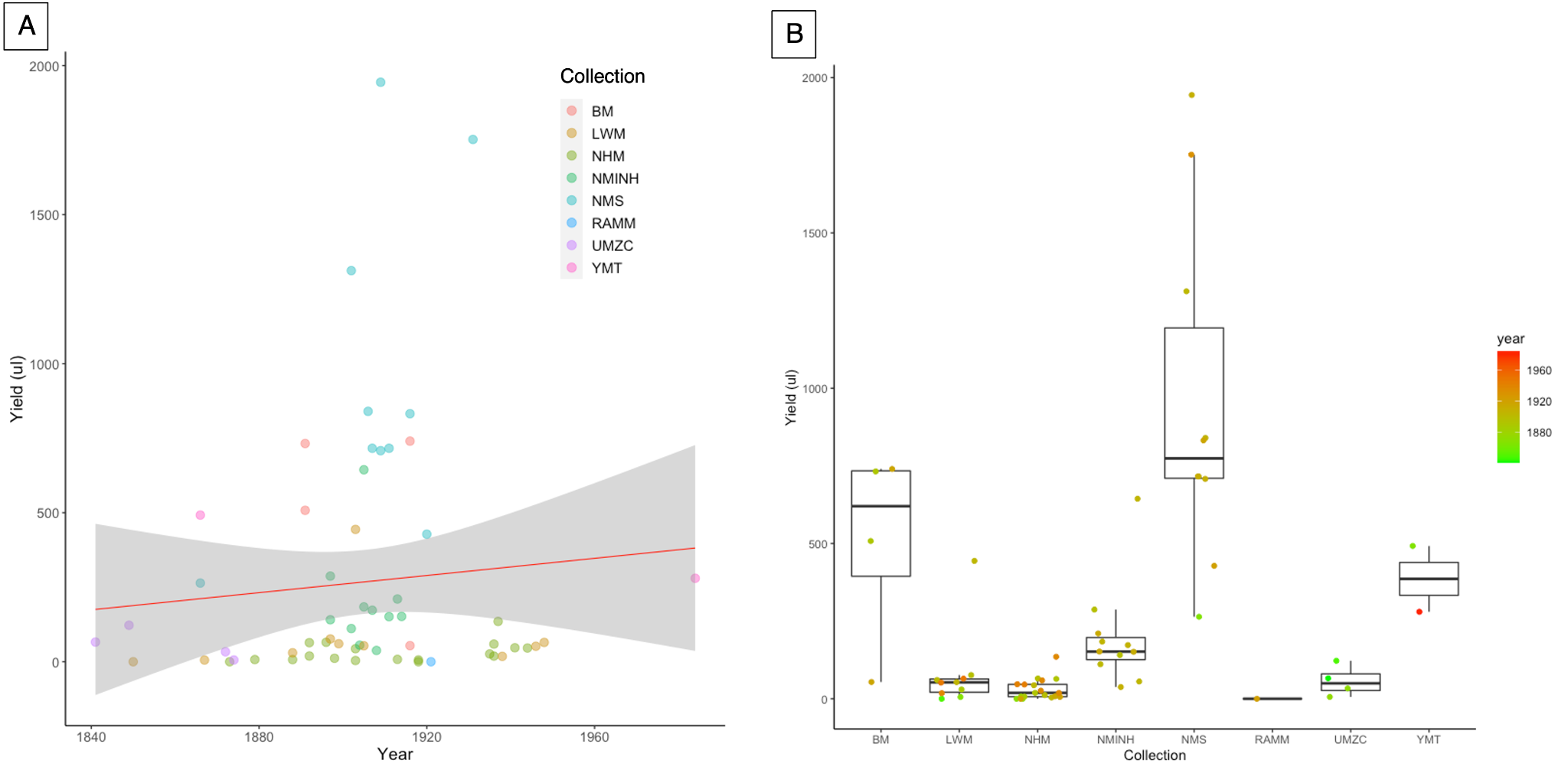


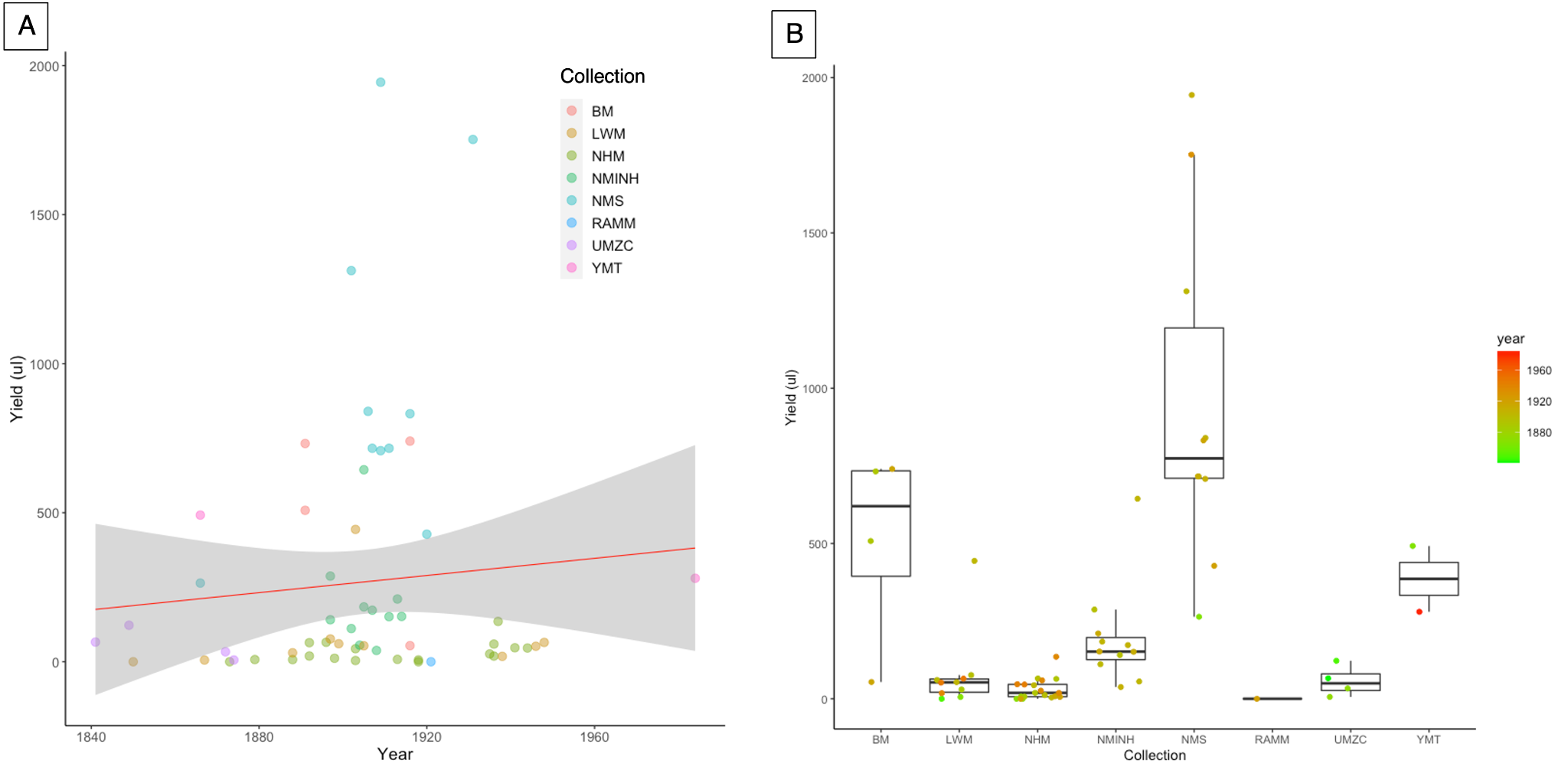


Fig, S1 relationship between yield and collection origin (A) and year (B) of museum specimens included in the Illumina sequencing. In plot A collection year is presented as colour gradient on overlain data point for context. Red line in plot A represents the trend line with the grey area shading representing 95% confidence intervals, with museum collections represented by different colour points. Museum collection codes on both figures are as follows; BM: Birmingham Museum, LWM: Liverpool World Museum, NHM: Natural History Museum (London), NMINH: National Museum of Ireland, NMS: National Museum of Scotland, RAMM: Royal Albert Memorial Museum,UMZC; Cambridge University Museum of Zoology, YMT: York Museum Trust.


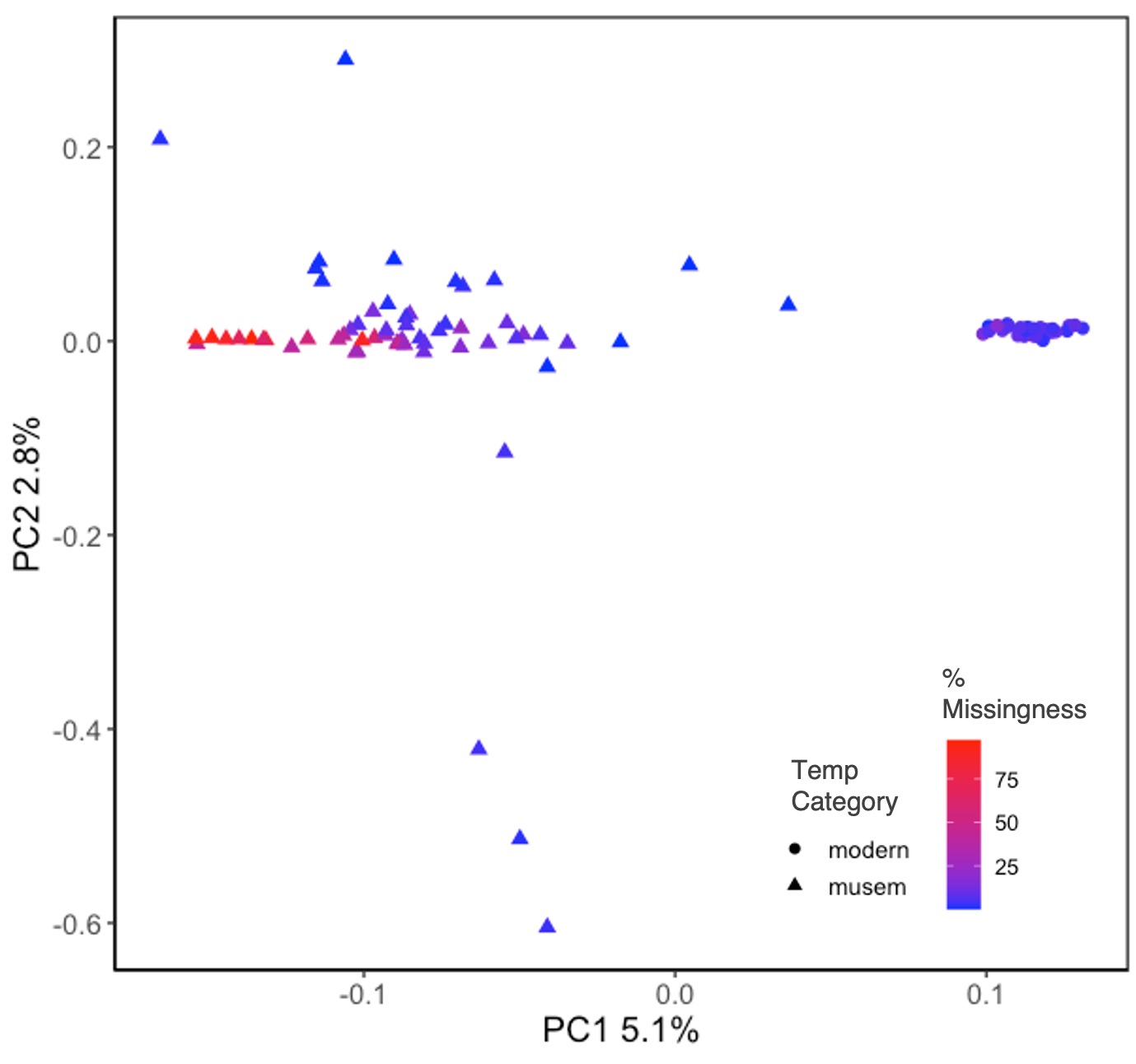


Fig, S2 PCA of all samples (historic and modern) from the downsampled data set with relative missingness (%) presented as colour ramp.


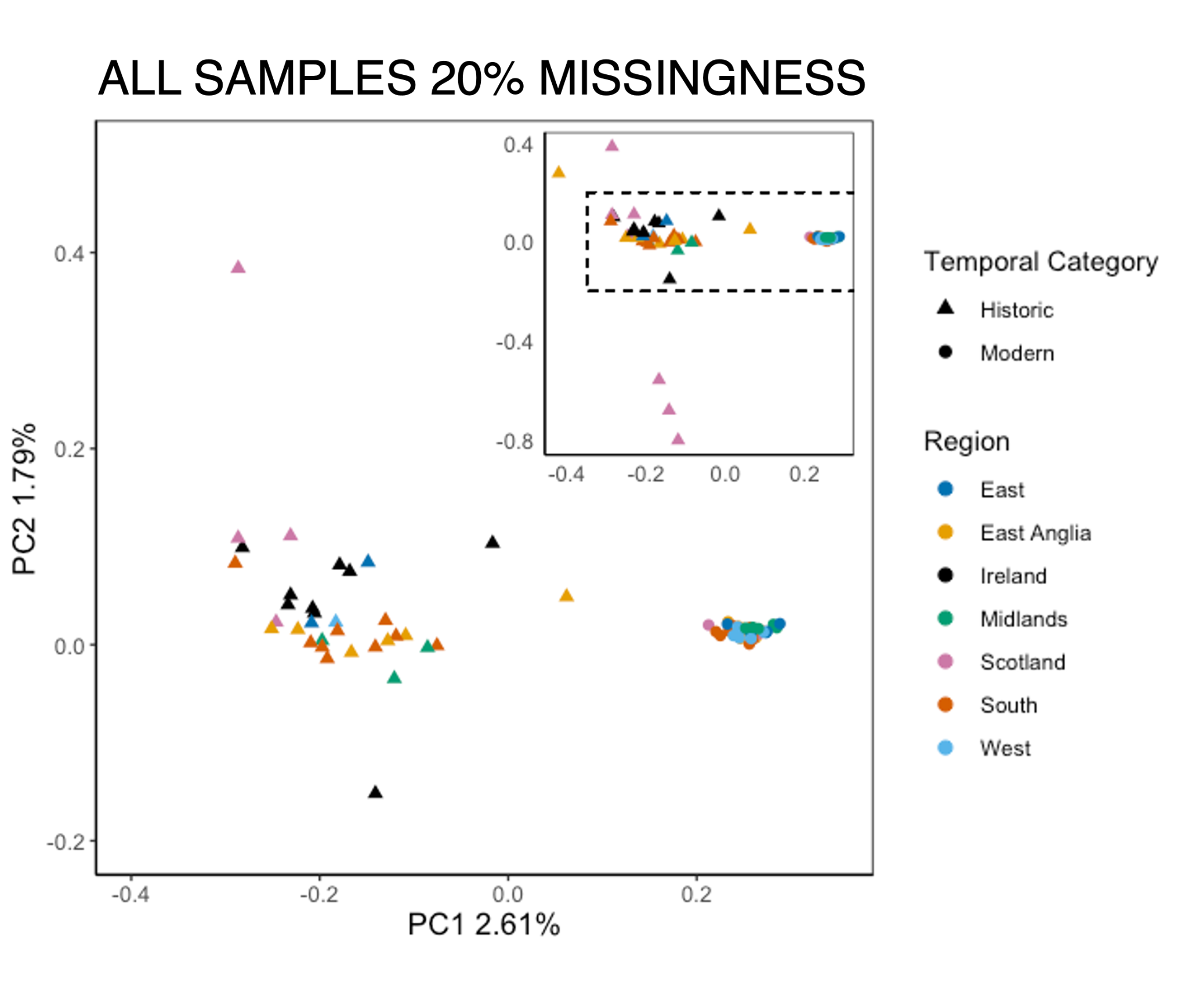


Fig, S3 PCA of all samples (historic and modern) from the downsampled data set with samples with a stringent 80% individual missingness filter applied (n = 61 individuals). The main plot is a cropped subplot of the embedded plot (top right), which shows all samples. The dashed boxes in the embedded plot shows the cropped area presented in the main plot. The plot has been cropped to remove the effect of strongly differentiated individuals on interpreting the genetic structure. Regional groupings (coloured triangles/circles) are presented as 95% confidence ellipses where clustering allows.


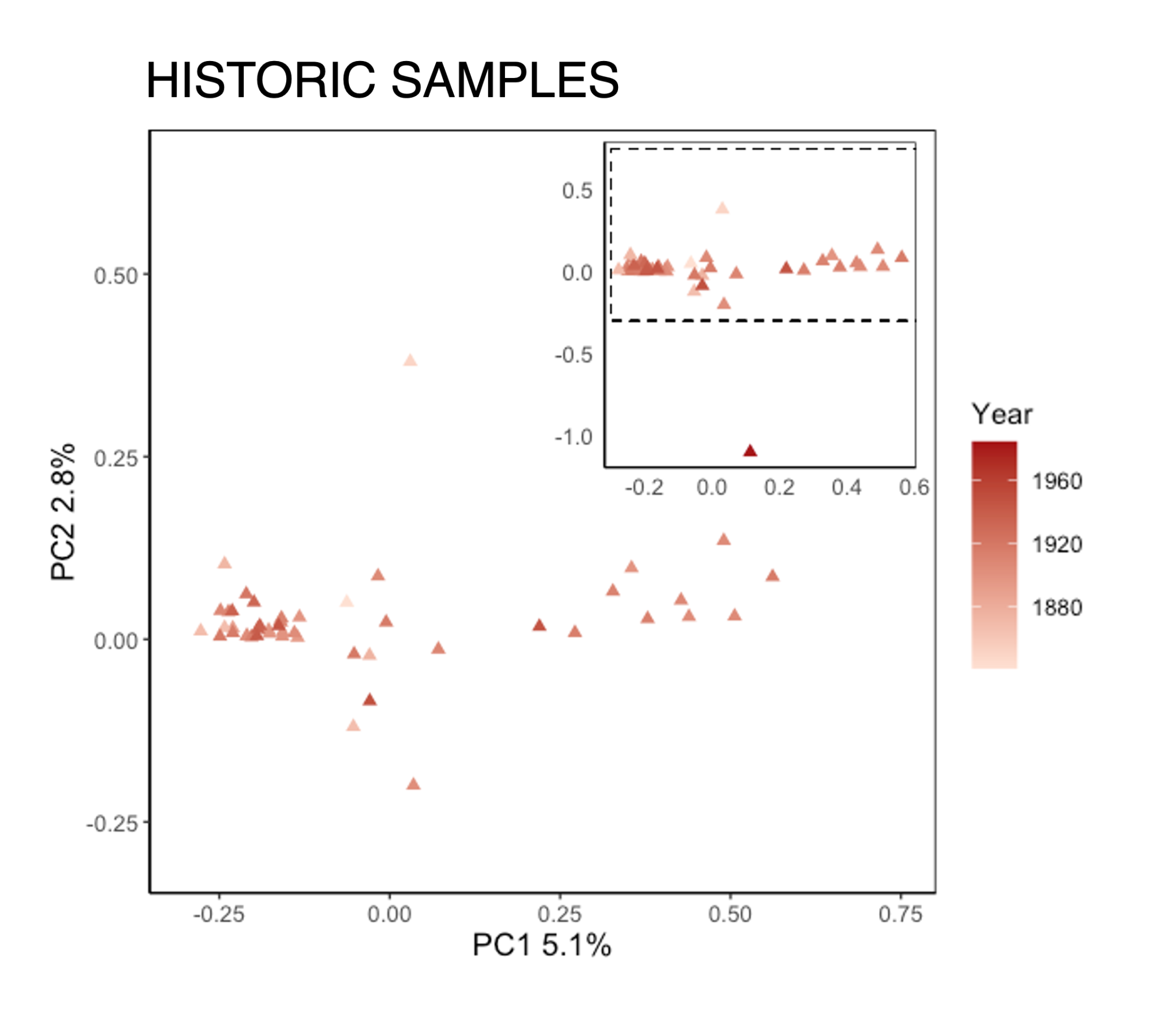


Figure S4 PCA biplots of genetic similarity calculated from down sampled filtered genotype likelihoods from historic samples, with colour ramp coding for sampling year.. Individuals with >50% missingness (n= 15) have been removed. The main plot is a cropped subplot of the embedded plot (top right), which shows all samples. The dashed boxes in the embedded plot shows the cropped area presented in the main plot. The plot has been cropped to remove the effect of strongly differentiated individuals on interpreting the genetic structure.


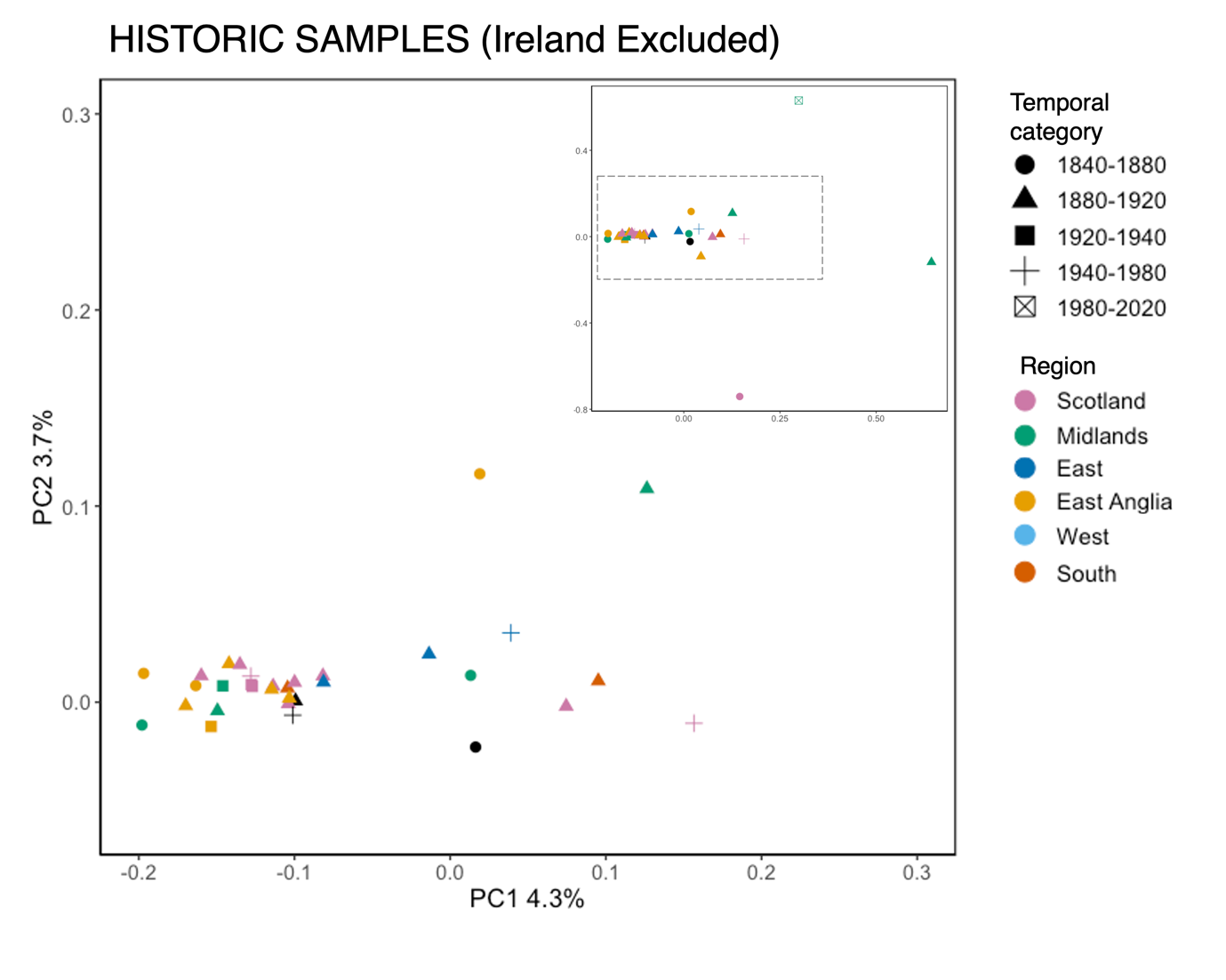


Figure S5 PCA biplots of genetic similarity calculated from down sampled filtered genotype likelihoods from historic samples with Irish samples removed. Individuals with >50% missingness (n= 15) have been removed. The main plot is a cropped subplot of the embedded plot (top right), which shows all samples. The dashed boxes in the embedded plot shows the cropped area presented in the main plot. The plot has been cropped to remove the effect of strongly differentiated individuals on interpreting the genetic structure. Regional groupings (coloured triangles) are presented as 95% confidence ellipses where clustering allows.


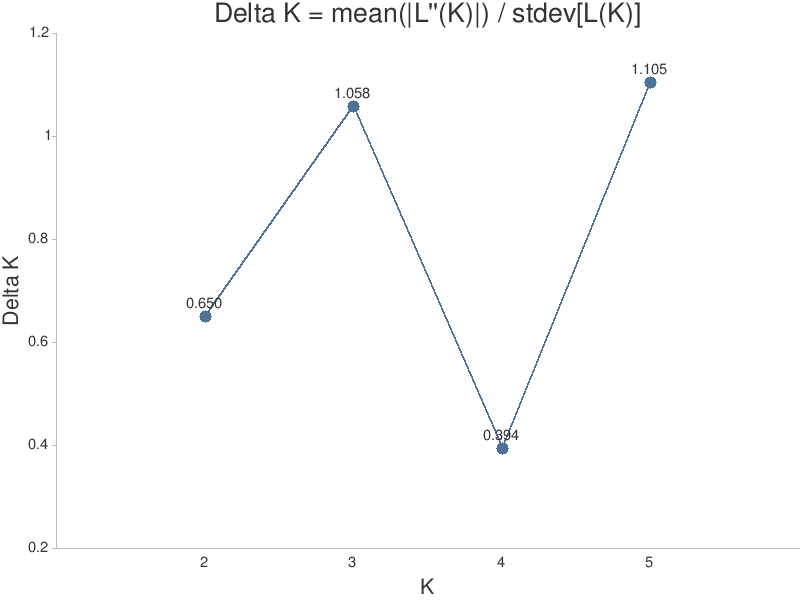


Fig, S6 Optimal (Delta) *K* for admixture analysis, where *K* = the number of sub or ancestral populations from which the total population is comprised. Figure generated from CLUMPAK optimal *K* analysis.


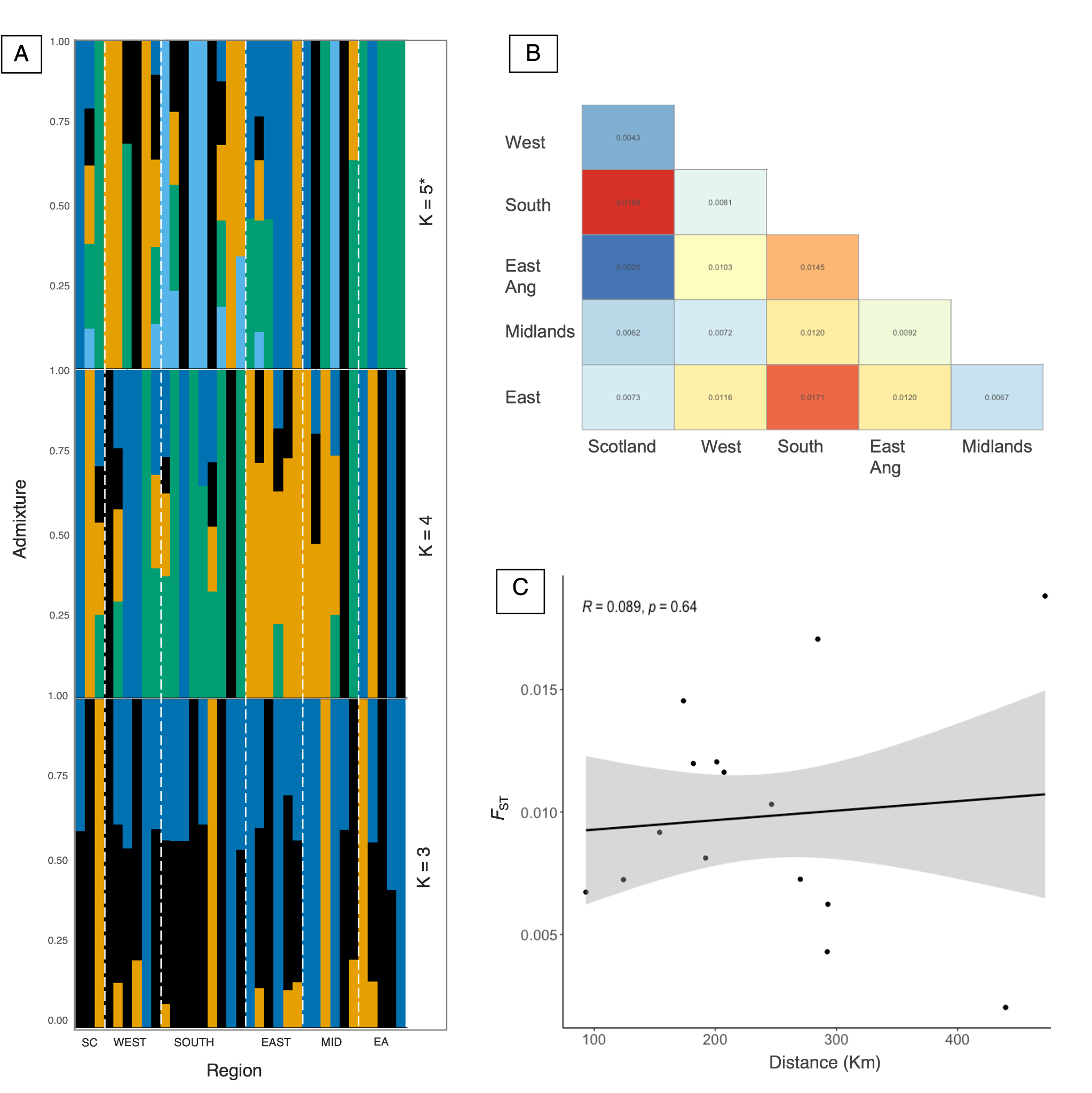


Figure S7 Genetic structure in modern samples. A) Admixture plot generated from NGSadmix for *K* = 3 – 5 (number of genetic clusters, coloured bars represent proportion of an individual's ancestry to *K* groups. EA = East Anglia, Mid = Midlands, Sc = Scotland. * = optimal *K* as per CLUMPAK; B) Pairwise weighted *F_ST_* values for each regional grouping, colour ramp (blue – red) indicates increasing population differentiation (*F*_ST_); C) Isolation by distance plot, showing correlation between pairwise *F_ST_* and pairwise spatial distances between region centroids.


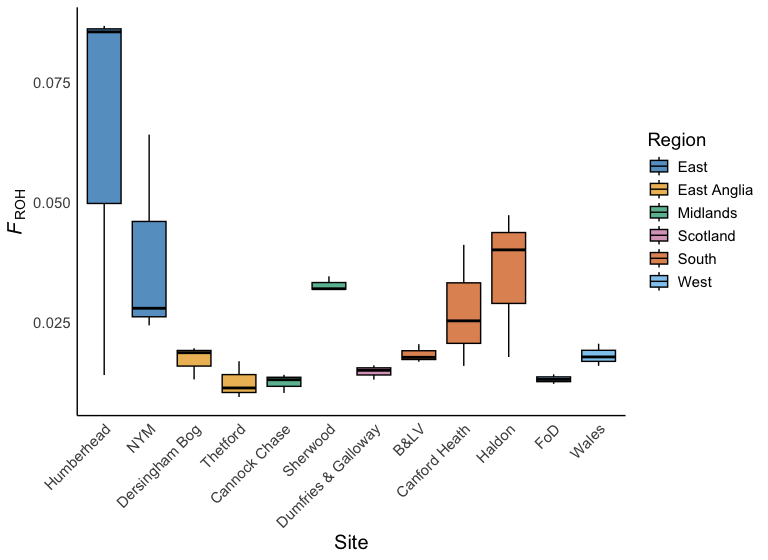


Fig S7 site specific *F*_ROH_ values within 100kb windows in modern samples grouped by regional categories (colour). Boxes represent median (midline) first and third quartiles and whiskers reflect range of values. Site abbreviations; Humberhead = Humberhead Peatlands, NYM = North Yorkshire Moors, Thetford = Thetford Forest, Sherwood = Sherwood Forest, B&LV = Bourley and Long Valley, Haldon = Haldon Forest Park, FoD = Forest of Dean, Wales = North Wales (n = 1), Mid Wales (n = 2).

**References**

Billerman, S. M. and Walsh, J. (2019). Historical DNA as a tool to address key questions in avian biology and evolution: A review of methods, challenges, applications, and future directions. Molecular Ecology Resources, 19 (5), pp.1115–1130. doi:10.1111/1755-0998.13066.

Irestedt, M., Thörn, F., Müller, I. A., Jønsson, K. A., Ericson, P. G. P. and Blom, M. P. K. (2022). A guide to avian museomics: Insights gained from resequencing hundreds of avian study skins. Molecular Ecology Resources, 22 (7). doi:10.1111/1755-0998.13660
